# Proteomic Analysis of Human Serum Proteins Adsorbed onto Collagen Barrier Membranes

**DOI:** 10.3390/jfb15100302

**Published:** 2024-10-09

**Authors:** Siddharth Shanbhag, Niyaz Al-Sharabi, Katarina Fritz-Wallace, Einar K. Kristoffersen, Dagmar Fosså Bunæs, Mario Romandini, Kamal Mustafa, Mariano Sanz, Reinhard Gruber

**Affiliations:** 1 Department of Clinical Dentistry, Faculty of Medicine, University of Bergen, 5009 Bergen, Norway; 2 Department of Immunology and Transfusion Medicine, Haukeland University Hospital, 5009 Bergen, Norway; 3 Department of Periodontology, Faculty of Dentistry, University of Oslo, 0455 Oslo, Norway; 4 Proteomics Unit of University of Bergen (PROBE), University of Bergen, 5009 Bergen, Norway; 5 Department of Clinical Medicine, University of Bergen, 5009 Bergen, Norway; 6 ETEP Research Group, University Complutense of Madrid, 28040 Madrid, Spain; 7 Department of Oral Biology, University Clinic of Dentistry, Medical University of Vienna, 1090 Vienna, Austria; 8 Austrian Cluster for Tissue Regeneration, 1200 Vienna, Austria; 9 Department of Periodontology, School of Dental Medicine, University of Bern, 3010 Bern, Switzerland

**Keywords:** guided bone regeneration, guided tissue regeneration, mass spectrometry, collagen membranes, protein extraction

## Abstract

Collagen barrier membranes are frequently used in guided tissue and bone regeneration. The aim of this study was to analyze the signature of human serum proteins adsorbed onto collagen membranes using a novel protein extraction method combined with mass spectrometry. Native porcine-derived collagen membranes (Geistlich Bio-Gide^®^, Wolhusen, Switzerland) were exposed to pooled human serum in vitro and, after thorough washing, subjected to protein extraction either in conjunction with protein enrichment or via a conventional surfactant-based method. The extracted proteins were analyzed via liquid chromatography with tandem mass spectrometry. Bioinformatic analysis of global profiling, gene ontology, and functional enrichment of the identified proteins was performed. Overall, a total of 326 adsorbed serum proteins were identified. The enrichment and conventional methods yielded similar numbers of total (315 vs. 309), exclusive (17 vs. 11), and major bone-related proteins (18 vs. 14). Most of the adsorbed proteins (n = 298) were common to both extraction groups and included several growth factors, extracellular matrix (ECM) proteins, cell adhesion molecules, and angiogenesis mediators involved in bone regeneration. Functional analyses revealed significant enrichment of ECM, exosomes, immune response, and cell growth components. Key proteins [transforming growth factor-beta 1 (TGFβ1), insulin-like growth factor binding proteins (IGFBP-5, -6, -7)] were exclusively detected with the enrichment-based method. In summary, native collagen membranes exhibited a high protein adsorption capacity in vitro. While both extraction methods were effective, the enrichment-based method showed distinct advantages in detecting specific bone-related proteins. Therefore, the use of multiple extraction methods is advisable in studies investigating protein adsorption on biomaterials.

## 1. Introduction

The rapid adsorption of blood proteins onto implanted biomaterials is a critical event that effectively translates the structure and composition of a foreign surface into a biologically recognizable one to which the host cells can respond [1,2]. Since cells rely on specific proteins for anchorage and extracellular signaling, the composition of the adsorbed protein layer is a crucial mediator of early cellular behavior, ultimately determining healing outcomes. In bone regeneration, early colonization of a biomaterial by mesenchymal progenitors and osteoblasts, followed by their differentiation and biosynthetic activity, is crucial [1,3,4]. Therefore, the proteins that adsorb onto bone biomaterials must support these cellular activities. Indeed, the adsorption of specific blood plasma or serum proteins on bone substitutes [5,6] and titanium implant surfaces [7,8], may correlate with their long-term clinical success [9,10].

Bioabsorbable collagen membranes are the most commonly used naturally derived membranes for guided tissue/bone regeneration (GTR/GBR), usually in combination with a bone graft or substitute [11,12,13,14,15]. These membranes are biologically favorable because collagen is the principal component of connective tissues, providing structural support and facilitating cell–matrix communication. Moreover, collagen offers several additional features that make it suitable for GBR applications, such as resorbability, low immunogenicity, and the ability to incorporate biological agents [14,16,17]. Recent attempts to functionalize membranes with extrinsic growth factors enhanced GBR outcomes in vivo [18,19,20,21]. Furthermore, experimental data from rodent models indicate that collagen membranes modulate the activity of host cells (osteoblasts, endothelial and inflammatory cells) locally within the defect microenvironment, thereby promoting GBR [14,16,22].

Quantitative proteomics is widely used to identify and compare the expression levels of large numbers of proteins in biological samples [23]. Using methods such as liquid chromatography with tandem mass spectrometry (LC-MS/MS), it is possible to obtain detailed and quantitative data on the proteome of a biological sample [24,25,26,27]. In the context of bone regeneration, previous studies have assessed the adsorption of serum or plasma proteins on bone substitutes [5,6,28]. However, to our knowledge, the profile of serum proteins adsorbed onto collagen membranes is unknown. Moreover, no previous studies have addressed the “dynamic range” challenge of serum and plasma proteins, which limits the reliable detection of “low-abundance” proteins, such as growth factors and cytokines [26]. In serum, only 10 proteins constitute ~90% of the entire proteome, and another 12 proteins comprise ~90% of the remaining fraction. These “high-abundance” proteins can mask the detection of several low-abundance proteins, which constitute only <1% of the total serum proteome. It is essential to identify these low-abundance proteins, as they are potentially biologically relevant [26]. Possible strategies to overcome this challenge include (a) the depletion of high-abundance proteins or (b) the enrichment of low-abundance proteins, of which the latter method is considered more suitable for quantitative proteomic analysis [26,29].

Although the proteomic composition of native collagen membranes has previously been analyzed [30], no studies have investigated the adsorption of blood/serum proteins on these membranes. If proteins are adsorbed on the membranes relative to their abundance in human serum, an enrichment step might be necessary to identify the low-abundance proteins of interest. Therefore, the objective of this preliminary study was to characterize the profile of human serum proteins adsorbed onto collagen barrier membranes using LC-MS/MS with or without enrichment for low-abundance proteins.

## 2. Materials and Methods

### 2.1. Membrane Preparation

A bi-layered, non-cross-linked porcine-derived collagen membrane (25 × 25 mm; Bio-Gide^®^, Geistlich Pharma, Wolhusen, Switzerland) was used in this study. Native membranes without serum served as controls. Following local ethical approval (AIT-69993), pooled human serum from three healthy volunteer donors (23–46 years; two males, one female) was obtained from the blood bank at Haukeland University Hospital, Bergen, Norway. Membrane samples were incubated with 2 mL of pooled serum at 37 °C for 1 h with intermittent shaking [31]. After incubation, the supernatants were removed and the membranes were thoroughly washed with phosphate-buffered saline (PBS; Invitrogen, Waltham, MA, USA) five times for 5 min each with shaking to remove loosely bound proteins. The samples were then left to dry and stored at –20 °C until further use.

### 2.2. Protein Extraction

Protein extraction from the membranes was performed using the following two “in-solution” methods: (1) a novel method based on the enrichment of low-abundance serum proteins (n = 3) and (2) a conventional surfactant-based method without enrichment (n = 3). For the first method, the recently introduced ENRICH-iST^®^ (EN) platform (PreOmics GmBH, Martinsried, Germany) was used. According to the manufacturer, the novelty of this method is the “unbiased enrichment of lower abundant plasma and serum proteins by 50% to more than 100%… in a cost-effective, fast 5-h workflow”, optimized for LC-MS/MS analysis (https://www.preomics.com/products/enrich-ist, accessed on 3 October 2024). For the second method, the RapiGest^®^ SF (RG) surfactant (Waters Inc., Milford, MA, USA), previously used for the LC-MS/MS analysis of native collagen membranes [30], was employed. Both methods followed the manufacturers’ protocols with the following modifications optimized during preliminary experiments. For the EN method, membranes were incubated with 80 μL of “binding buffer” (EN-BIND, PreOmics GmBH) ON at 4 °C with shaking. For the RG method, membranes were incubated with 80 μL of 0.5% RG for 10 min at RT. After incubation, both EN- and RG-treated samples were sonicated for 10 min in a water bath. The supernatants containing extracted proteins (membranes removed) were transferred to fresh tubes and processed downstream according to the respective EN and RG manufacturers’ protocols.

### 2.3. LC-MS/MS

The samples were analyzed using label-free LC-MS/MS quantitation, as previously described [32]. Approximately 0.7 ug of tryptic peptides dissolved in 2% acetonitrile and 0.5% formic acid was injected into an Ultimate 3000 RSLC system connected to an Exploris 480 mass spectrometer equipped with an EASY-spray nano-electrospray ion source (all from Thermo Fisher), and MS2 spectra were acquired using data-dependent acquisition (DDA). Additional details of the LC-MS/MS setup are provided in the Supplementary Materials. The proteomics data have been deposited to the Proteome-Xchange Consortium via the PRIDE partner repository (https://www.ebi.ac.uk/pride/; accessed on 7 August 2024) with the dataset identifier PXD054665.

### 2.4. Bioinformatic and Statistical Analysis

LC-MS/MS data from experimental (membranes with serum) and control samples (membranes without serum, serum alone) were searched using Proteome Discoverer software (version 2.5.0.400; Thermo Fisher Scientific Inc., Waltham, MA, USA) against the human SwisProt database (downloaded in October 2022, including 20,401 sequences), the *Sus scrofa* UniProt database (downloaded in March 2024, including 46,173 sequences), and a list of common contaminants. Further data analysis was performed using Perseus software (version 2.0.9.0; Max-Planck-Institute of Biochemistry, Martinsreid, Germany) [33]. A four-step filtration strategy was applied to select the relevant proteins, based on (a) human origin, (b) detection of ≥2 peptides, (c) detection in all three replicates, and (d) co-detection in human serum samples but not in native membrane samples (without serum). Relevant human gene ontology (GO) terms were retrieved from the QuickGO database (EMBL-EMI, Cambridgeshire, UK; www.ebi.ac.uk/QuickGO/, accessed on 15 August 2024). GO profiling was performed using the g: Profiler software (version e111_eg58_p18_30541362) [34] based on the molecular function (MF), biological process (BP), and cellular component (CC) databases. Functional enrichment analysis (FEA) was performed using the FunRich open access tool, which applies the hypergeometric test with Bonferroni correction for *p*-values (*p* < 0.05) [35]. Differentially expressed proteins (DEPs) between EN and RG methods were identified based on relative abundances using a two-sided Student’s *t*-test in combination with permutation-based correction for multiple hypothesis testing (false discovery rate; FDR = 0.05).

## 3. Results

### 3.1. Global Profiling of Adsorbed Serum Proteins

**Figure 1:**
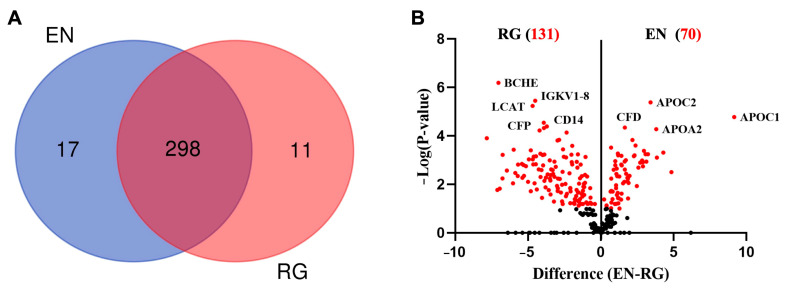
Comparison between the two protein extraction methods. (**A**) Venn diagram illustrating the numbers of common and exclusive proteins between the ENRICH (EN) and RapiGest (RG) methods. (**B**) Volcano plot showing differentially expressed proteins (DEPs) (adjusted p-value) vs. magnitude of expression change (log2-fold change) between RG (n = 131 DEPs) and EN (n = 70 DEPs).

**Figure 2:**
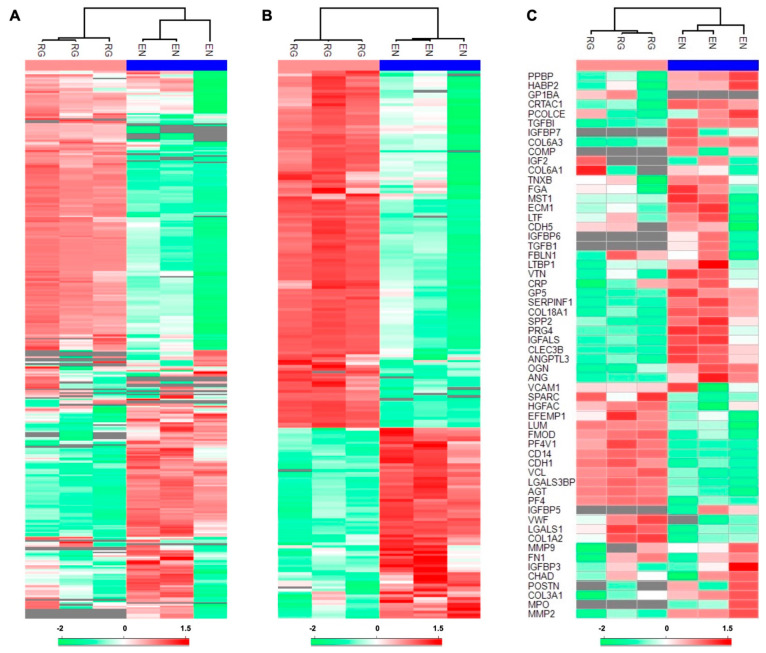
Hierarchical clustering analysis of adsorbed serum proteins in the ENRICH (EN) and RapiGest groups (RG). (**A**) All adsorbed proteins. (**B**) Differentially expressed proteins. (**C**) Bone-related proteins. Intensity values (protein abundances) are represented as colors ranging from low (green) to high (red); n = 3 technical replicates in each group.

Based on the inclusion criteria, a total of 326 human serum proteins were identified. Of these, 298 proteins were commonly identified using both EN and RG methods, with EN revealing slightly more exclusive proteins (n = 17) compared to the RG group (n = 11) (Figure 1A; Supplementary Tables S1–S3). The quantitative analysis of the common proteins revealed 201 DEPs, of which 131 were significantly more abundant in RG and 70 in the EN groups (Figure 1B and Figure 2A–C; Supplementary Tables S4 and S5). The profiles of pooled serum alone (human proteins) and native membranes alone (porcine proteins) were also analyzed (Supplementary Tables S6 and S7).

### 3.2. Functional Analysis of Adsorbed Serum Proteins

**Figure 3:**
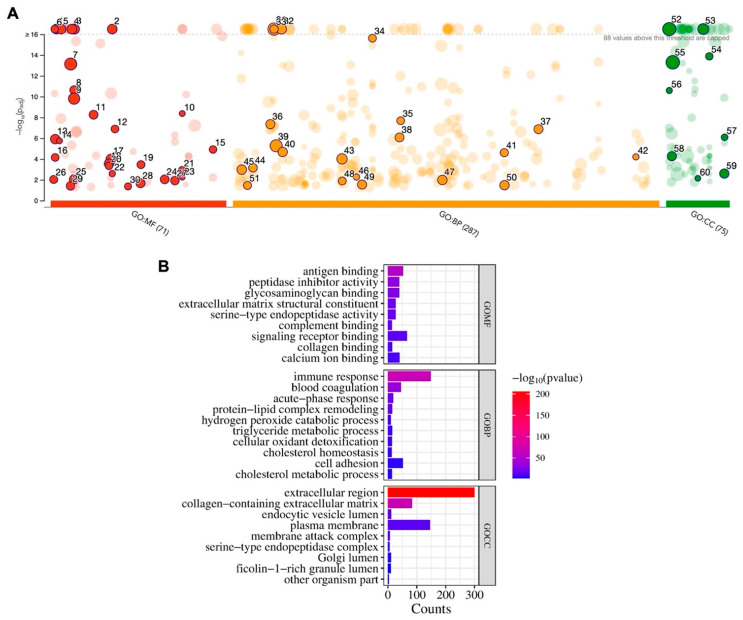
GO analysis of serum proteins adsorbed on collagen membranes. (**A**) Results of enrichment analysis presented in the form of a Manhattan plot, where the *X*-axis shows the functional terms grouped by the color code of the source database used, and the *Y*-axis shows the enrichment adjusted p-values in a negative decimal logarithm scale. Dots in the graph indicate all enriched terms meeting the significance criterion of *p* < 0.05, while highlighted dots represent terms filtered by the criterion of top 10 terms. (**B**) The graphs show detailed results of the enriched terms highlighted in the Manhattan plot along with the statistical significance [−log10(*p*-value)] and number of common proteins (counts) belonging to the enriched term, according to molecular function (MF), biological process (BP), and cellular component (CC) categories.

Since the majority of proteins were identified using both extraction methods, a single GO profiling and FEA was performed on all adsorbed serum proteins. The GO analysis revealed the enrichment of MF (n = 71 terms), BP (n = 287), and CC categories (n = 75) (Figure 3A). Among the top 10 enriched terms, several were related to wound healing, i.e., MF (“extracellular matrix structural constituent”, “collagen binding”, “calcium ion binding”), BP (“immune response”, “blood coagulation”, “cell adhesion”) and CC (“extracellular region”, “collagen-containing extracellular matrix”) (Figure 3B). Specific BP terms associated with bone healing and regeneration are presented in Table 1.

**Figure 4:**
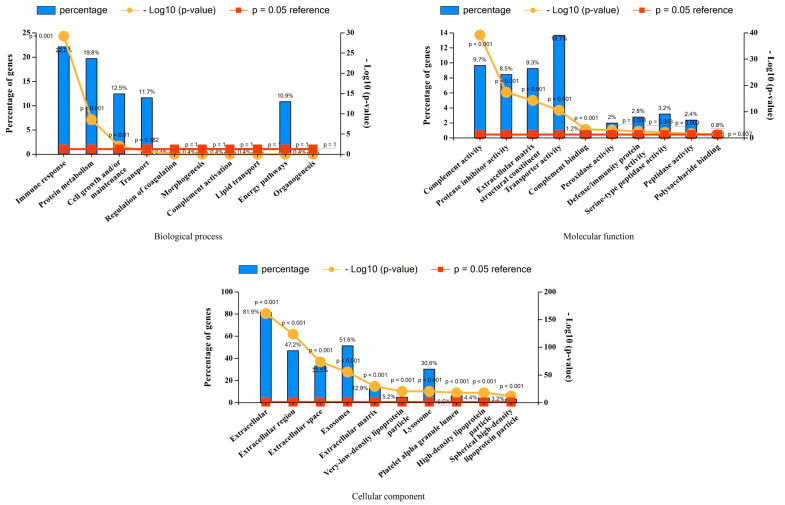
Functional enrichment analysis of adsorbed serum proteins. Graphs showing the percentage representation and significance levels (*p*-values) of the top 10 enriched groups under “Biological processes”, “Molecular function”, and “Cellular components” categories. *p* < 0.05 indicates statistically significant enrichment.

The FEA revealed significant enrichment (*p* < 0.05) in several BP (n = 3), MF (n = 10), and CC categories (n = 10). Many of these categories such as BP (“immune response”, “cell growth and/or maintenance”), MF (“complement activity”, “ECM structural constituent”), and CC (“extracellular region/space/matrix”, “exosomes”) were relevant in the context of bone regeneration (Figure 4).

### 3.3. Identification of Major Bone-Related Proteins

The adsorbed serum proteins included several key candidates involved in bone regeneration (Table 2). These included major ECM proteins [collagens (COL1, COL3, COL6), metalloproteinases (MMP2, MMP9), osteoglycin (OGN), periostin (POSTN), osteonectin (SPARC), tetranectin (CLEC3B), ECM protein 1 (ECM1)], cell adhesion molecules [cadherins (CDH1, CDH5), fibronectin (FN1), vitronectin (VTN)], growth factors [transforming growth factor-beta (TGFβ) family, insulin-like growth factor (IGF) family, hepatocyte growth factor (HGF) family], and angiogenesis mediators [angiogenin (ANG), angiopoietin-related protein (ANGPTL3), vascular cell adhesion protein 1 (VCAM1)]. Additionally, several apolipoproteins (APOA1, APOB, APOC, APOD, APOE) and proteins related to soft-tissue healing [pigment epithelium-derived factor (SERPINF1), keratinocyte differentiation-associated protein (KRTDAP)] were detected. Although not included in the main dataset, four additional proteins with only one detected peptide [but with a peptide spectrum match (PSM) = 3] were identified, namely C-C motif chemokines 5 (CCL5) and 14 (CCL14), endothelial protein C receptor (PROCR), and platelet-activating factor acetylhydrolase (PLA2G7), also relevant for wound healing (Supplementary Table S8).

While most proteins were identified using both EN and RG methods, a few were exclusively identified in the EN group [IGF-binding proteins (IGFBP5, IGFBP6, IGFBP7), TGFβ1, myeloperoxidase (MPO)] or the RG group [platelet glycoprotein Ib alpha chain (GP1BA)] (Table 2). Slightly more bone-related proteins were detected among the DEPs in the EN group compared to the RG group (n = 18 vs. 14). In general, the EN method yielded greater numbers of identified peptides and detection coverage (%) for bone-related proteins (Table 2).

## 4. Discussion

The objective of this in vitro study was to characterize the profile of human serum proteins adsorbed onto collagen barrier membranes using LC/MS combined with a novel protein extraction method aimed at enriching low-abundance serum proteins. A total of 326 proteins were identified, including growth factors, angiogenesis mediators, ECM components, and cell adhesion molecules, all relevant in the context of bone regeneration. The FEA revealed a significant enrichment of ECM, exosomes, and cell growth components. A major finding was that the enrichment-based and conventional surfactant-based methods were comparable in overall efficacy, with the EN method demonstrating a slight advantage in detecting specific proteins (e.g., TGFβ1, IGFBP-5, -6, -7) relevant to bone regeneration adsorbed onto collagen membranes.

The adsorption of proteins from biological fluids onto the surface of a biomaterial occurs immediately after implantation, and to a large extent, determines its biocompatibility and bioactivity [1,2,4]. Even though not fully understood, electrostatic and hydrogen bonding were proposed as the underlying mechanisms of protein adsorption [36]. As certain proteins can elicit specific cellular responses, it is important to study the composition of the adsorbed proteins, including growth factors, to predict the potential in vivo behavior of a biomaterial [4]. Several proteins important for bone regeneration were found to be adsorbed onto collagen membranes herein (Table 2), thereby supporting previous findings. For instance, growth factors such as TGFβ1 [37] and BMPs [38,39] are reported to effectively adsorb to collagen. Indeed, recombinant BMP2 adsorbed onto a collagen sponge serving as a delivery vehicle is available as an FDA-approved product [38]. These observations do not rule out that members of the TGFβ superfamily exclusively bind to collagen. With regard to IGFBPs (-5, -6 and -7), although these are not recognized as binding to collagen, they can directly adsorb to proteoglycans [40], which are ECM components that already exist in the native collagen membrane [30]. Moreover, apolipoprotein A-I, the main apolipoprotein of high-density lipoproteins, effectively adsorbs to collagen and glycosaminoglycans [41]. Collagenases (MMP2, MMP9), which are involved in bone remodeling [42], were also identified herein and their presence can be explained by the binding to type I collagen fibrils, which is not a substrate for either of the enzymes [43], and fibronectin [44]. Indeed, the presence of certain non-collagenous matrix proteins in the membranes, such as leucine-rich repeat proteoglycans [30], may also have facilitated serum protein adsorption. Further research is needed to understand how specific proteins bind to collagen membranes, directly or indirectly. Understanding these principles is the basis for tailoring biomaterial properties to promote selective protein adsorption, thereby enhancing regenerative outcomes [45].

Although previous studies have not directly investigated the adsorption of serum proteins on collagen-based membranes in the context of GBR, similar studies have been conducted on bone substitute materials [5,6,46,47,48]. For instance, calcium phosphate-based (CaP) ceramics, known to be highly osteoconductive, adsorb over 500 different serum proteins [5]. Consistent with the present findings, these proteins are related to cellular adhesion, differentiation, and ECM components. Moreover, since the protein adsorption capacity of a material is related to its morphological and physiochemical properties (surface topography, roughness, wettability, etc.), modifying these properties may affect protein adsorption [49,50,51]. For example, the addition of silica to CaP ceramics can enhance protein adsorption [28] and consequently, in vivo performance [52]. Previous studies have comprehensively characterized the morphological and physiochemical properties of native collagen membranes, including Bio-Gide^®^. These membranes have demonstrated favorable surface porosity (>60%), roughness, and wettability, which potentially support protein adsorption [53,54,55,56]. These favorable physiochemical properties reportedly translate to excellent in vitro cytocompatibility (cell adhesion) and in vivo biocompatibility of collagen membranes [22,53]. It is reasonable to assume that these effects are mediated, at least partly, via the adsorption of regulatory proteins. However, it has not been studied whether certain physiochemical modifications of collagen membranes, e.g., cross-linking, may alter early protein adsorption and subsequent cellular responses and in vivo outcomes. The EN method might be helpful in this respect for future studies of protein adsorption.

Previous studies have predominantly used surfactants, most commonly sodium dodecyl sulfate (SDS), to extract adsorbed proteins from biomaterials. However, SDS can interfere with LC-MS analyses by reducing sensitivity, thereby necessitating a removal step [57]. The RG product used in the present study is a surfactant similar to SDS, but is easily removed by an acidification step prior to LC-MS. Indeed, RG has previously been used for the proteomic analysis of native Bio-Gide^®^ membranes [30]. Additionally, the dynamic range of plasma and serum proteins, which refers to variations in concentrations by up to 12 orders of magnitude [58], has not been adequately addressed so far. High-abundance proteins, such as albumin, immunoglobulins, etc., may “mask” the detection of low-abundance proteins, including growth factors, cytokines, etc., which potentially play a role in bone regeneration [25,58]. Masking may distort the “true” identification of adsorbed proteins. Therefore, in the present study, we adapted a novel enrichment method (EN) designed to detect these low-abundance proteins for the extraction of adsorbed proteins from the collagen membranes and compared it to a surfactant-based (RG) protein extraction.

We anticipated differences between the more “subtle” EN and the more “aggressive” RG methods. Surprisingly, both methods identified a similar number of proteins, with slightly more total (315 vs. 309) and exclusive proteins (17 vs. 11) detected in the EN group. Notably, proteins exclusively identified in the EN group included key regulators of bone (IGFBP5, -6, -7, TGFβ1) [59,60] and soft-tissue healing (KRTDAP) [61]. Despite the identification of considerably more DEPs in the RG vs. EN group (131 vs. 70), several growth factors (TGFβI, IGFALS, SERPINF1) and ECM components (MMP2, SPP2, CLEC3B, OGN) involved in bone regeneration were significantly more abundant in the EN group. Additionally, apolipoproteins, such as APOA1 and APOB, linked to bone metabolism [62,63], were more abundant in the EN group, while APOE, which is strongly linked to bone formation [64], was similarly detected in both groups. In general, the EN method identified more peptides (N) and protein detection coverage (%) for bone- and healing-related proteins as compared to the RG method, thereby providing distinct advantages in terms of detecting potentially clinically relevant proteins adsorbed on collagen membranes.

Early research of GTR/GBR highlighted the importance of membranes for space maintenance over a bone defect and stabilization of the blood clot, which would in turn promote vascularization, osteogenic cell supply, and new bone formation [65,66,67,68]. Recent in vivo data further suggest that collagen membranes not only act as “passive” barriers but actively promote cellular and molecular events during healing [69,70]. In this context [71], previous studies have suggested favorable clot formation [72], angiogenesis [73,74,75], and cellular activity in relation to Bio-Gide^®^ membranes [76,77] after relatively short exposure/implantation times. In the present study, several coagulation- and platelet-related serum proteins were detected on the collagen membranes, including platelet glycoprotein V (GP5), which is required for platelet adhesion and aggregation [78]. Collagen itself induces platelet adhesion, aggregation, and activation, which are relevant for coagulation and subsequent chemotaxis [79]. Additionally, angiogenesis mediators (e.g., ANG, ANGPTL3, ECM1) [71] and cell adhesion molecules (e.g., fibronectin, vitronectin, laminin, and cadherin) [1,3,80], were identified. These proteins, particularly fibronectin, support osteoblast differentiation [81]. Likewise, several growth factors, such as IGF2, IGFBP5, and TGFβ1, which are established regulators of osteoblast differentiation [82], and bone-specific ECM molecules such as OGN (bone turnover) [83], SPARC (cell-ECM interaction, mineralization) [84], CLEC3B (mineralization) [85], POSTN (osteoblast differentiation) [86], etc., were detected. Previous studies in rodent models [69,70] have demonstrated that barrier membranes actively contribute to GBR by modulating cellular and molecular events, e.g., gene expression, in the defect microenvironment. The present data suggest, albeit hypothetically, that these beneficial effects may at least partly be mediated via the bioactive proteins adsorbed on the collagen membranes upon in vivo implantation.

The present FEA also revealed the enrichment of the exosomes component, which is relevant given the emerging role of extracellular vesicles (EVs) in periodontal and bone regeneration [87,88,89]. In addition to soluble proteins, serum is known to contain EVs, which mediate cellular responses via paracrine mechanisms [90]. A post hoc analysis further confirmed that at least 10 of the “top 100 EV proteins” according to Vesiclepedia [91] were identified among the adsorbed proteins. EVs, including serum EVs, have gained significant attention due to their high regenerative efficacy and promising clinical potential [92]. Recently, our group has reported the use of Bio-Gide^®^ membranes as scaffolds for the delivery of MSC-EVs in rat calvaria defects; preliminary in vitro analyses showed efficient adhesion and infiltration of EVs into the membrane body [93]. Further studies are warranted on microparticle and other osteopromotive factors related to the adsorption on collagen membranes to potentially promote clinical outcomes.

As most “proof of concept” studies, the present study has limitations. (i) It must be acknowledged that serum prepared ex vivo does not mimic an in vivo coagulum in structure and composition. Therefore, the interactions of collagen membranes with other blood components (plasma proteins, cells, etc.) encountered during the early stages of GTR/GBR remain elusive. (ii) Pooled serum from three donors was used herein to minimize donor-related variation. The inclusion of individual donors might reveal donor-specific differences in protein adsorption. (iii) No functional assay was performed to confirm the bioactivity of the adsorbed serum proteins, which is a challenge, since native collagen membranes already affect cellular activity in vitro and cell culture/seeding requires serum supplementation [21,94]. (iv) Another limitation is the high sequence homology between several human and pig proteins. Previous proteomic analyses of native collagen membranes have revealed, in addition to different collagens, several other ECM molecules (e.g., BGN, LUM, OGN, etc.), which may also influence cellular activity [30,95,96]. Nevertheless, given the filtering conditions for peptides and proteins, as well as the high accuracy of the mass spectrometer, it is rather unlikely that proteins were misassigned, despite the high homology between human and porcine proteins. Moreover, candidate proteins were checked manually for possible sequence similarity in both organisms and potentially excluded. (v) Finally, only one type of collagen membrane, widely used in clinical GBR applications, was tested herein. Further studies are needed to investigate the patterns of protein adsorption on other types of membranes, e.g., cross-linked membranes, and biomaterials, e.g., bone substitutes.

While current preclinical data may support the evidence for a bioactive role of barrier membranes [16,70], the clinical translation of these findings is merely speculative and requires further research. Moreover, whether these promotive effects are related to the membranes’ physiochemical properties and whether the modification of these properties may enhance these effects still needs to be studied [45]. With regard to protein adsorption, an open question remains as to whether barrier membranes serve as a reservoir for bioactive molecules (e.g., growth factors) even after the replacement of the blood clot. The identification of specific soft-tissue healing markers such as SERPINF1 and KRTDAP warrants further investigation into the effects of collagen membranes on mucosal healing.

## 5. Conclusions

In summary, the enrichment-based (EN) and surfactant-based (RG) methods were comparable in the extraction and identification of adsorbed serum proteins from native collagen membranes. The EN method showed distinct advantages in detecting specific bone- and healing-related proteins. A functional analysis revealed a significant enrichment of the ECM, cell growth, and exosome components involved in bone regeneration. These data further support the current evidence for an active role of collagen membranes in modulating cellular and molecular events during GBR [15]. Further research is needed to assess the impact of physiochemical modifications, such as cross-linking, on protein adsorption and to determine whether adsorbed proteins induce functional responses in vitro and in vivo.

## Figures and Tables

**Table 1:** Representation of selected gene ontology (GO) terms and corresponding numbers (n) and percentages (%) of identified proteins related to wound healing and bone regeneration.

GO Term	Term ID	Term Size (n)	Proteins Identified (n)	%
wound healing	GO:0042060	411	42	10.22
blood coagulation	GO:0007596	248	37	14.92
platelet aggregation	GO:0070527	53	5	9.43
angiogenesis	GO:0001525	381	17	4.46
cell adhesion	GO:0007155	1333	38	2.85
extracellular matrix organization	GO:0030198	315	21	6.67
osteoblast differentiation	GO:0001649	156	5	3.21
bone development	GO:0060348	212	9	4.25
bone mineralization	GO:0030282	64	5	7.81

**Table 2:** Bone healing-related serum proteins identified by EN or RG methods.

		EN				RG			
ID	Name	Det.	Cov. %	Pep. n	Unique n	Det.	Cov. %	Pep. n	Unique n
**Coagulation**									
PPBP	Platelet basic protein	Y *	38	5	5	Y	38	5	5
GP5	Platelet glycoprotein V	Y *	16	5	5	Y	-	-	-
PF4	Platelet factor 4	Y	19	2	1	Y *	27	5	4
VWF	von Willebrand factor	Y	1	2	2	Y *	2	3	3
GP1BA	Platelet glycoprotein Ib alpha chain	N				Y	5	3	3
**Extracellular matrix **									
MMP2	Matrix metallopeptidase-2 (72 kDa type IV collagenase)	Y *	28	14	14	Y	3	1	1
MMP9	Matrix metalloproteinase-9 (92 kDa type IV collagenase)	Y	9	5	5	Y	-	-	-
COL3A1	Collagen alpha-1(III) chain	Y *	3	4	2	Y	1	2	1
COL18A1	Collagen alpha-1(XVIII) chain	Y *	4	5	5	Y	-	-	-
COL6A3	Collagen alpha-3(VI) chain	Y *	14	33	19	Y	5	12	1
COL1A2	Collagen alpha-2(I) chain	Y	7	7	2	Y *	7	8	4
COL6A1	Collagen alpha-1(VI) chain	Y	6	5	2	Y	-	-	-
PRG4	Proteoglycan 4	Y *	9	11	11	Y	10	10	10
SPP2	Secreted phosphoprotein 24	Y *	10	2	2	Y	6	1	1
CLEC3B	Tetranectin	Y *	64	10	10	Y	54	12	9
HABP2	Hyaluronan-binding protein 2	Y *	15	10	10	Y	25	10	10
EFEMP1	EGF-containing fibulin-like extracellular matrix protein 1	Y	10	4	4	Y *	26	8	8
FMOD	Fibromodulin	Y	9	3	3	Y *	6	2	2
LGALS1	Galectin-1	Y	-	-	-	Y *	20	2	2
LGALS3BP	Galectin-3-binding protein	Y	11	4	4	Y *	31	12	12
LUM	Lumican	Y	30	8	4	Y *	41	14	14
SPARC	Osteonectin	Y	4	1	1	Y *	26	4	4
CHAD	Chondroadherin	Y	23	4	4	Y	-	-	-
OGN	Osteoglycin (Mimecan)	Y *	36	10	4	Y	19	6	1
ECM1	Extracellular matrix protein 1	Y	33	13	13	Y	27	10	10
FBLN1	Fibulin-1	Y	18	10	10	Y	28	14	14
LTF	Lactotransferrin	Y	18	11	11	Y	-	-	-
CRTAC1	Cartilage acidic protein 1	Y *	38	15	15	Y	24	10	10
POSTN	Periostin	Y	5	2	2	Y	2	1	1
PCOLCE	Procollagen C-endopeptidase enhancer 1	Y	20	7	7	Y	13	3	3
TNXB	Tenascin-X	Y	4	8	8	Y	1	3	3
COMP	Cartilage oligomeric matrix protein	Y	8	4	3	N			
**Growth factors**									
TGFB1	Transforming growth factor beta-1 proprotein	Y	7	2	2	N			
TGFBI	Transforming growth factor-beta-induced protein ig-h3	Y *	27	13	13	Y	23	10	10
LTBP1	Latent-transforming growth factor beta-binding protein 1	Y	4	5	5	Y	-	-	-
IGF2	Insulin-like growth factor II	Y	4	1	1	Y	-	-	-
IGFALS	IGF-binding protein complex acid labile subunit	Y *	35	16	16	Y	35	15	15
IGFBP3	IGF-binding protein 3	Y	10	3	3	Y	21	4	4
IGFBP6	IGF-binding protein 6	Y	10	2	2	N			
IGFBP7	IGF-binding protein 7	Y	10	2	2	N			
IGFBP5	IGF-binding protein 5	Y	7	2	2	N			
HGFAC	Hepatocyte growth factor activator	Y	13	5	5	Y *	15	6	6
MST1	Hepatocyte growth factor-like protein	Y	15	10	10	Y	18	8	8
**Angiogenesis**									
ANG	Angiogenin	Y *	18	3	3	Y	20	2	2
ANGPTL3	Angiopoietin-related protein 3	Y *	17	5	5	Y	-	-	-
AGT	Angiotensinogen	Y	31	9	9	Y *	44	13	13
VCAM1	Vascular cell adhesion protein 1	Y	-	-	-	Y *	5	2	2
**Cell adhesion**									
VCL	Vinculin	Y *	5	3	3	Y	-	-	-
VTN	Vitronectin	Y *	29	13	13	Y	32	14	13
CDH1	Cadherin-1	Y	2	2	2	Y *	-	-	-
CDH5	Cadherin-5	Y	5	3	3	Y	2	2	2
FGA	Fibrinogen alpha chain	Y	16	10	10	Y	7	4	4
FN1	Fibronectin	Y	33	51	23	Y	41	64	64
**Inflammation**									
CD14	Monocyte differentiation antigen CD14	Y	20	5	5	Y *	45	13	13
CRP	C-reactive protein	Y	14	3	3	Y	15	2	2
MPO	Myeloperoxidase	Y	5	3	3	N			
**Lipoproteins **									
APOA1	Apolipoprotein A–I	Y *	85	40	37	Y	84	44	42
APOB	Apolipoprotein B	Y *	62	229	229	Y	51	192	189
APOD	Apolipoprotein D	Y	34	6	6	Y *	35	8	8
APOE	Apolipoprotein E	Y	50	16	16	Y	50	18	16
**Soft-tissue healing**									
SERPINF1	Pigment epithelium-derived factor	Y *	67	25	18	Y	63	20	14
KRTDAP	Keratinocyte differentiation-associated protein	Y	26	2	2	N			

EN, ENRICH method; RG, RapiGest method; Det., detected; Y, yes; N, no; Cov., coverage (%); Pep., peptides (n, number); Unique, unique peptides (n); * significantly greater abundance; -, not detected in all three replicates.

## Data Availability

Additional data are presented in the supplementary files and can be made available upon reasonable request. The proteomics data have been deposited to the Proteome-Xchange Consortium via the PRIDE partner repository (https://www.ebi.ac.uk/pride/; accessed on 7 August 2024) with the dataset identifier PXD054665.

## Supplementary Materials

The following supporting information can be downloaded at: https://www.mdpi.com/article/10.3390/jfb15100302/s1, Supplementary methods (File S1), Supplementary Tables S1–S8 (File S2). Table S1: All identified human serum proteins adsorbed on collagen membranes. Table S2: Exclusive proteins in the EN group. Table S3: Exclusive proteins in the RG group. Table S4: DEPs in the EN group. Table S5: DEPs in the RG group. Table S6: Proteins identified in pooled human serum. Table S7: Proteins identified in native collagen membranes. Table S8: Relevant proteins with only a single identified peptide.
