# Supplementary material for: Proteomic Analysis of Human Serum Proteins Adsorbed onto Collagen Barrier Membranes
Source: J Funct Biomater. 2024 Oct 9;15(10):302. doi: 10.3390/jfb15100302 (PMC11508515; doi:10.3390/jfb15100302)
Supplement: Supplementary file 1 [file jfb-15-00302-s001.zip › File S1.pdf]

Supplementary file

Additional methods: LC-MS/MS

LC run (195 min)
Peptides were separated during a biphasic ACN gradient from two nanoflow UPLC pumps (flow rate of 200 nl/min) on a 50 cm analytical column (PepMap RSLC, 50cm x 75 µm ID EASY-spray column, packed with 2µm C18 beads). Solvent A and B were 0.1% TFA (vol/vol) in water and 100% ACN respectively. The gradient composition was 5%B during trapping (5min) followed by 5-8%B over 1 min, 8–25%B for the next 124min, 25-35%B over 30 min, and 35–85%B over 5min. Elution of very hydrophobic peptides and conditioning of the column were performed during 10 minutes isocratic elution with 85%B and 15 minutes isocratic conditioning with 5%B. Instrument control was through Thermo Scientific SII for Xcalibur 1.6.

High field asymmetric waveform ion mobility spectrometry (FAIMS)
The FAIMS Pro interface performs gas-phase fractionation, enabling preferred accumulation of multiply charged ions to maximize the efficiency of data-dependent acquisition (DDA) routines and increase proteome coverage. Short-ion residence time in the FAIMS Pro interface electrode assembly enables use of multiple CV settings in a single run to increase proteome coverage.

DDA with FAIMS
Peptides eluted from the column were detected in the Exploris 480 Mass Spectrometer with FAIMS enabled using three compensation voltages (CVs), -45V, -65V and -80V respectively, and "Advanced Peak Determination" on. During each CV, the mass spectrometer was operated in the DDA-mode (data-dependent-acquisition) to automatically switch between one full scan MS and MS/MS acquisition. Instrument control was through Orbitrap Exploris 480 Tune 3.1 and Xcalibur 4.4. The cycle time was maintained at 1.2s/CV (-45 and -65) or 0.8s/CV (-80). MS spectra were acquired in the scan range 375-1500 m/z with resolution R = 120 000 at m/z 200, automatic gain control (AGC) target of 3e6 and a maximum injection time (IT) at auto (depending on transient length in the orbitrap). The most intense eluting peptides with charge states 2 to 6 were sequentially isolated to standard target value (AGC, usually 1e5) or a maximum IT of 75 ms in the C-trap, and isolation width maintained at 1.6 m/z (quadrupole isolation), before fragmentation in the Higher-Energy Collision Dissociation (HCD). Fragmentation was performed with a normalized collision energy (NCE) of 30 %, and fragments were detected in the Orbitrap at a resolution of 15,000 at m/z 200, with first mass fixed at m/z 120. One MS/MS spectrum of a precursor mass was allowed before dynamic exclusion for 30s with "exclude isotopes" on. Lock-mass internal calibration was not enabled.

Ionsource parameter
The spray and ion-source parameters were as follows. Ion spray voltage = 2000V, no sheath and auxiliary gas flow, and capillary temperature = 275 °C.
